# Young Children Exposed to Intimate Partner Violence Describe their Abused Parent: A Qualitative Study

**DOI:** 10.1007/s10896-016-9856-5

**Published:** 2016-09-13

**Authors:** Karin Pernebo, Kjerstin Almqvist

**Affiliations:** 1 Department of Psychology, Linnaeus University, Växjö, Sweden; 2 Department of Research and Development, Region Kronoberg, Box 1223, S-351 12 Växjö, Sweden; 3 Department of Social and Psychological Sciences, Karlstad University, Karlstad, Sweden

**Keywords:** Child, Children witnessing intimate partner violence, Domestic violence, Qualitative research, Children’s accounts

## Abstract

The negative impact of intimate partner violence (IPV) begins early in the child’s relationship with a caregiver. Children’s relationships with, and internal working models of, abused parents have rarely been documented. The aim of this study was to collect and interpret young children’s accounts of their abused parent. Interviews were conducted with 17 children aged 4 to 12 years who had witnessed IPV. Thematic analysis identified three main themes and seven sub-themes: “Coherent accounts of the parent” (sub-themes of “general benevolence”, “provision of support, protection, and nurture”, and “parental distress”); “Deficient accounts of the parent” (“vague accounts” and “disorganized narrations”); and “The parent as a trauma trigger” (“avoidance” and “breakthrough of intrusive memories and thoughts”). The results indicate these children may hold integrated, deficient, or blocked internal representations of an abused parent, and they illustrate the benefit of including young children as informants in research.

Witnessing violence and threats against a caregiver during the earliest years of life is associated with severe effects on children’s health and development. Resulting issues can include symptoms of psychological distress, behavior disorders, disturbances in self-regulation, difficulties in social interaction, and disorganized attachment (Evans et al. 2008; Holt et al. 2008; Scheeringa and Zeanah 1995). The negative impact of intimate partner violence (IPV) on the child begins very early in the relationship with the caregiver, before problem behaviors and deficits in the child’s general well-being are noted (Levendosky et al. 2003). These effects may be more serious in younger than in older children (Fantuzzo et al. 1997; Levendosky et al. 2013). Younger children are more completely dependent on their caregivers than older children, not only for physical care, but also for emotional closeness and safety required for normal neurological, psychological, and social development. This dependence may contribute to their vulnerability to the effects of witnessing violence against their caregiver (Levendosky et al. 2013; Schore 2013; Siegel 2012).

Being subjected to IPV influences mothers’ parenting in various ways. Some mothers abdicate their role or become impulsive and harshly punitive towards their children, while others compensate for the violence by becoming more engaged and competent parents (George and Solomon 2008; Levendosky et al. 2003). Intimate partner violence creates a context in which the abused parent’s availability to the child and predictable behavior are jeopardized, creating a situation that threatens to leave the child without sufficient support in the areas of physical and emotional regulation (Levendosky et al. 2011; Schore and Schore 2008). Not being able to rely upon either the abusive or the abused parent for protection, support, and emotional regulation may undermine the child’s confidence in the parents’ availability (Kobak and Madsen 2008; Zeanah et al. 2011). The child may lose both the sense of being cared for and nurtured and the trust in the caregivers’ capacity to provide support and protection (Swanston et al. 2014). Parents who appear helpless and fearful, who have abdicated their parental role, or seem incapable of protecting themselves and the child may instill fear in the child and risk putting the child in a reversed role in relation to the abused parent.

Witnessing IPV has been shown to affect the attachment relationship and the child’s inner representations of both the abusive and abused parent (Sternberg et al. 1994; Sternberg et al. 2005). Children’s inner representations and developing internal working models of their caregivers and the world are closely related to early relational experiences and have been found to influence their future expectations and behaviors (Bretherton and Munholland 1999). Children exposed to IPV show a heightened prevalence of disorganized attachment and of later controlling attachment patterns (Levendosky et al. 2011; Levendosky et al. 2003; Zeanah et al. 2011; Zeanah et al. 1999). Disorganized attachment has been shown to predict role-reversed relationships between young children and their mothers (Macfie et al. 2008; Van Ijzendoorn et al. 1999), to hamper the child’s capacity for emotional regulation and ability to establish future relations with significant others (Kogan and Carter 1996; Schore 2013; Sroufe 2005), and to be a strong risk factor for future disturbances including behavior problems, symptoms of post-traumatic stress disorder, and dissociation (Sroufe 2005; Van Ijzendoorn et al. 1999; Zeanah et al. 2011).

A considerable proportion of children exposed to IPV show persisting symptoms that indicate a need for treatment, even after the abused parent separates from the perpetrator (Grych et al. 2000). Several evaluations of interventions for children exposed to IPV against a caregiver have shown that including the abused parent in treatment and targeting the relationship between the child and caregiver is associated with positive effects (Levendosky et al. 2003; Lieberman et al. 2006; Lieberman et al. 2005; Macfie et al. 2008; Stover et al. 2009). In therapy, professionals meet parents and children with a focus on the child-parent dyad, on interactions between the caregiver and the child, and on the child’s internal working model of the attachment relationship. Internal working models are dyadic and changeable, with a dual potential to both promote positive change and simultaneously increase risk for the development of additional symptoms and difficulties. This makes internal working models one of the most important targets for interventions in treatment (Bowlby 1980; Sroufe 2005).

Adjacent fields of research concerning how children describe a violent parent have returned complex results. Children who have witnessed IPV have been found to describe both positive and negative feelings about their violent fathers (Cater 2007; Staf and Almqvist 2015). Maltreated children tend to be more likely than others to develop negative representations of their parents, and their representations of mothers have been shown to be less integrated, less benevolent, and more punitive regardless of which parent is the perpetrator (Manashko et al. 2009; Sternberg et al. 2005). Some maltreated children, however, show an adaptive cognitive style and a tendency to idealize the caregiver by overstating their benevolence (Manashko et al. 2009).

Current understanding of how children experience their relationship with the abused parent stems mainly from theory, behavioral observations, parental reports, and the memories of teenagers or adults. The voices of children, young children in particular, are fairly absent in research (Spratling et al. 2012). The right of children to be heard in issues that concern them is central to the United Nations Convention on the Rights of the Child, and including children’s voices has been shown to improve acknowledgment of their needs and to optimize efforts and measures to meet those needs. Parental reports of children’s experiences, however, have shown low reliability (Appel and Holden 1998; Biering 2010; Lambert et al. 1998) and Øverlien (2010) argue that listening to children is necessary to gain insight into their own understandings. Children’s views may be neglected because of doubts about their capacity to report their experiences and express their opinions reliably (Day et al. 2006). However, a number of researchers have verified children’s capacity to retain and provide accurate, organized, verbally accessible, and valuable memories and experiences (Dockett and Perry 2007; Evang and Øverlien 2015; Fivush 1998; Hershkowitz et al. 2012).

There is a gap in the research into how young children describe their abused parent and their relationship with that parent. Qualitative research that emphasizes children’s perspectives and perceptions has the potential to provide information that is otherwise lacking and thus contribute to our understanding of children’s experience. Such knowledge would be helpful in designing, performing, and evaluating preventive interventions and treatment models for children and parents exposed to IPV. This qualitative study aimed to expand knowledge of how children exposed to IPV describe their abused parent.

## Method

### Participants

Interviews were conducted with 17 children, 10 girls and seven boys, from 4.5 to 12.3 years (M = 7.1 years, Med = 5.9 years). Background information was provided by the caregiver after consent and prior to the interview. All children spoke Swedish and lived in one of the two major urban areas in Sweden. All but two of the children were born in Sweden, but nine had at least one parent who was not native to Sweden. Thirteen children lived with the abused parent, three lived alternately with each parent, and one child lived in foster care. Swedish legislation generally grants separated parents the right to regular unsupervised overnight visits with the child in the parent’s home, but in this sample, nine of the children had no visits with their father. The children had all witnessed IPV against a parent**—**16 against the mother and one against the father. In 13 cases the perpetrator was the biological father, in two cases it was a new partner of the mother, in one case it was the biological mother, and in one case both the biological father and a new partner of the mother were perpetrators. The violence against the parent included psychological violence (e.g., threats, insults, and controlling behavior) and physical violence (e.g., slapping, pushing, kicking, choking, and sexual abuse). All parents except one reported that the physical violence against them had ceased, but six parents reported ongoing exposure to verbal offences and threats from the perpetrator. According to the parents, 12 of the children had been physically abused by the same perpetrator as the parent and five had not. At the time of the interview, seven parents of the physically abused children reported no ongoing violence against the children and the other five reported not knowing whether the violence had ceased or continued during visitations with the other parent.

### Procedure

The informants were chosen for their ability to contribute to the research question using purposive sampling. The children were recruited from two agencies for children exposed to domestic violence, and had all taken part in well-established manual-based group interventions for children exposed to IPV. Treatment length was 12 to 15 weekly sessions. All abused caregivers had received some treatment at the same agencies, either in group sessions or individually. Taking part in the support programs implied that the IPV had been revealed and acknowledged by the child and the abused parent. The parents considered IPV to be the main reason for taking part in the intervention and no formal trauma screening was performed.

During spring 2013, all parents of children taking part in group treatment at the agencies received written and verbal information about the study by staff members and were asked at their next appointment for consent to participate. All parents approached agreed to their child’s participation and provided background information on themselves and their child. The research interview was scheduled at the end of the intervention program and the parents were encouraged to inform their child about the study. The children received verbal information about the study at the time of the interview and were asked for their consent to participate. All children agreed to participate. Sixteen children were interviewed individually, and one child chose to have the mother in the room. During the interviews the children’s consent was seen as an ongoing process, and the children were able to influence their own participation in terms of the length of the interview and the depth of their responses (Dockett and Perry 2007, 2011). Children could pause, end the interview, or pass on a question at any time. All interviews took place at the agencies in March, May, and June 2013. The interviews lasted from 19 to 53 min and were conducted by the first author, an experienced child psychologist and psychotherapist.

Face-to-face interviews were conducted using a semi-structured interview guide. To establish rapport and to give the children an opportunity to practice being interviewed (Lamb and Brown 2006), the children were first asked about going to preschool or school or about something they liked doing at home. The interview then focused on the core request to talk about the abused parent and their relationship with that parent. Broad invitations and open questions were used, such as “Can you tell me about your mom?” and “Can you tell me about you and your dad?” Follow-up questions probed responses already given by the child as cues for further questioning, including such prompts as “Can you tell me more about that?” and “Can you explain what you mean?” In addition to answering the questions verbally the children were given the opportunity to illustrate their answers by drawing or using toys. The illustrations and toys informed the interview, but did not form part of the data for analysis. All interviews were recorded and transcribed verbatim, and only the verbal statements of the children were analyzed.

### Analysis

Thematic analysis as described by Braun and Clarke (2006) was used to identify, analyze, and report patterns in the data. The analysis was performed inductively using a contextual approach, which is considered appropriate when the aim is to describe, in context, how participants understand their experiences (Braun and Clarke 2006). The process was guided by the research question, with the aim of identifying, analyzing, and reporting the statements the children made about their abused parent. The process of analysis is described below; for a more detailed description of the phases of thematic analysis, see Braun and Clarke.

Each transcribed interview was read several times by the researcher. Notes were made of significant topics, and initial codes close to the content of the transcripts were made. Themes were then grouped within and across interviews. Coded extracts within each theme were read and compared to identify similarities and differences. Finally, grouping the themes and creating sub-themes resulted in three main themes and seven sub-themes. Main and sub-themes were checked against the original transcripts and adjustments were made if necessary. Each step of the analysis was first carried out independently by the first author and then revised in collaboration with the second author before the next step.

### Ethical Considerations

Research with traumatized and vulnerable children must be conducted with special attention to those children’s right to informed consent, the imbalance in power between researcher and child, possible conflicts of loyalty for the child, and the risk of discomfort or overwhelming experiences during the interview. These considerations were acknowledged in this study and steps were taken to recognize each child’s limits for participation, to gain approval of the child’s participation from the abused parent, to keep the focus of the interview on the research question, and to avoid encouraging the child to speak about the trauma itself. Although used in this paper for clarification, expressions such as “the abused parent” or the “abusive parent” were not used in the interviews with the children. When children spontaneously discussed their trauma the interviewer actively responded to validate their statements, but did not encourage further exploration of that topic. The interviews were carried out in a setting well-known to the child, and the child was reunited with the caregiver after the interview. Additional professional support was accessible if needed. The study was approved by the Regional Ethics Committee in Uppsala (Dnr 2012/246).

## Results

The analysis of the interviews with the children resulted in three themes with seven sub-themes (see Table 1). The themes are presented and illustrated by quotes from the children, who could contribute to more than one theme or sub-theme. Minor changes have been made to the quotes to protect the children’s privacy and to adjust for the translation from Swedish.

**Table 1:** Main themes and sub-themes from the interview analysis

Main themes	Sub-themes
Coherent accounts of the parent	General benevolence
	Provision of support, protection, and nurture
	Parental distress
Deficient accounts of the parent	Vague descriptions of parent
	Disorganized narratives
Parent as trauma trigger	Avoidance
	Breakthrough of intrusive memories and thoughts

### Coherent Accounts of the Parent

When asked to talk about their abused parent many of the children were able to provide nuanced descriptions. These children seemed to have access to integrated working models of the parent and a capacity to reflect on different aspects of the parent and of the child-parent relationship. They talked about the parent coherently by describing the parent’s resources and shortcomings. Three groups of parental qualities were described: general benevolence; provision of support, protection, and nurture; and parental distress.

#### General Benevolence

All children related things they were used to doing with the abused parent, talking about concrete activities and frequently about their shared joy during those activities. For many of the children the relationship with the caregiver seemed to be associated with a relaxed and happy mood. Several children repeatedly described the parent as good, kind, and considerate, and they expressed a sense of trust and safety in being with the parent. These children described a unique relationship with a feeling of mutuality and closeness.

Interviewer: Can you tell me about your mom?

Child: She is kind, and very cheerful… she is fun …

I: Yes? … can you explain, how?

C: Well, I mean she is really kind, because she helps all the time, if there is anything, and she is kind of helpful, and then she is, she is …

I: What did you just think about?

C: Um, I mean, we laugh together if something is funny or … (Girl, 9 years old)

I: Can you tell me about you and your mom?

C: … Well, you know, we kind of talk …

I: Uhuh? …

C: Yes …

I: Can you tell me anything else about your mom?

C: … She is fun … and kind and considerate.

I: Can you explain? … How do you notice that?

C: You notice that because she always asks if something was good and things like that … (Boy, 11 years old)

#### Provision of Support, Protection, and Nurture

The children described the parent as having capacities associated with the parenting functions of being older, wiser, stronger, and able to nurture, guide, and protect the child. Several children focused on the practical responsibilities taken by the parent such as cleaning the house, paying the rent, shopping, and preparing food. Others described how the parent nurtured them emotionally.

I: What can you tell about your mom?

C: She is very kind. Gives us healthy food.

I: Okay … and what more? Can you tell more?

C: She always gives me carrots at home, eh, when we are about to eat, before dinner. (Girl, 8 years old)

C [Explaining how his mother helps him with his homework]: She tells me when I do it right or wrong. When I am right she says “Good,” but when she says … when I’m wrong she says “Think again” […] I mean she doesn’t say “no, that’s wrong, do it again, you are no good.” I mean, I don’t want her to say things like that, I think she should say “Good” or “Stop, and think again.” I mean, I like her the way she is, when she says that. (Boy, 7 years old)

The children also talked about the parent as providing guidance through socialization and setting limits. Some children described their parent as fair, as someone who could give and allow freedom as well as restrict and set limits. Others said that they found it annoying but acceptable when their parent nagged them, while a few described the parent as angry and too harsh.

C: … I mean sometimes when I ask if someone [playmate] can come, then she [C’s mother] almost always says yes … But when we are going shopping … she says a lot of no … then she almost always says that it is not okay, then she says that you can come back later, when we are back home, and then you can play. (Boy, 7 years old)

C: … but it is very annoying to hear Mom nag.

I: Oh? What does she nag about then?

C: She nags us about having to take a shower, having to wash my hair even if I don’t want to.

I: Okay, what happens then?

C: Then it happens that … that I do it. (Girl, 5 years old)

I: Can you tell me about your mom?

C: She is … [clears his throat] … too strict.

I: Can you explain how you mean?

C: [Drinks water.] She nags a lot really, and it is … hmm, she gets pretty angry with [X].

I: Who?

C: [X], and sometimes with me. I mean my little sister. (Boy, 9 years old)

A few of the children mentioned that the parent cares for them by finding solutions, taking responsibility, and protecting them from physical violence.

C: As I said, they have explained what we should do, and Mom has said, “Yes it [the door] is locked, he can’t do anything,” and then I feel a little better now. (Boy, 9 years old)

#### Parental Distress

At times the children described the parent as distressed. Among these some portrayed the parent as highly aroused, showing heightened responsiveness or hyperactivity and stress, while others described the parent as sad, unresponsive, or absent. In both cases the parent was described as unavailable to the child.

C: She felt stressful, stressful.

I: Ah. .. how is do you notice that? What is she like then?

C: She becomes angry because we don’t listen.

I: Okay, so she is stressful and angry…

C: Yes, because we don’t listen, it is serious. (Boy, 5 years old)

C: Oh, but I … I can’t think of anything we do … I do not come up with anything … [stops drawing, groans, knocks herself on the head, whimpers]

I: Are you thinking?

C: Oh yes …

I: When you knock on your head, do more thoughts come then?

C: No … ah, I can’t think of anything … she only sits by the computer all the time; she doesn’t do anything with me …

I: Your mom?

C: … almost … I only watch TV. (Girl, 8 years old)

### Deficient Accounts of the Parent

In contrast to the coherent and reflective accounts of the parent, the interviewed children also sometimes had difficulty describing and reflecting upon the abused parent. At these times the children seemed to have difficulty verbalizing their experience of the parent and their accounts were either vague or disorganized and often difficult to follow, indicating flat or shattered working models of the parent. For some of the children, these difficulties were general and persisted during the whole interview, while others showed a more inconsistent pattern with a mixture of coherent and deficient descriptions of their parent.

#### Vague Descriptions of Parent

Several of the children responded to questions about the abused parent by giving flat and vague descriptions. Some focused on describing or drawing the parent’s clothing or other details and others had difficulties coming up with any description of the parent as a person or of sharing any common activities; they expressed a sense of absence, shortage, and passivity in relation to the abused parent.

C: She is … her name is [X].

I: Yes? …

C: She has hair like me …

I: Okay, uhuh …

C: And she has … she has … actually … hmm … black sweater …

I: Uhuh… okay …

C: And she has … no, I don’t know more. (Girl, 5 years old)

I: … Can you tell me about you and your mom?

C: Um, um … no … I, I cannot.

I: What are you thinking about?

C: I’m thinking about when … I can’t think of anything we have done together that much, eh …

I: Can you think of one thing?

C: … Oh, yes, now I think about what we have done together: we have come here. (Girl, 8 years old)

#### Disorganized Narratives

Some of the children talked about the abused parent in a disorganized manner; they were often physically restless and frequently shifted focus; their statements could be fragmentary and contradictory, and their accounts were often difficult to follow. A few children described a relationship with the parent in which the roles and hierarchy shifted between a clear parent-child relationship, an equal playmate-like relationship, and occasionally a relationship in which the roles were reversed.

I: Can you tell me about you and your mom?

C: Yes. Mom, you know she … [Stands up and move towards some toys.]

I: Come and sit down …

C: Later we are going to play. Do you know how long it takes to travel to Italy?

I: Um …

C: Three hours.

I: Okay, three hours. But tell me about you and your mom?

C: Okay, my mom.

I: Yes …

C: Sometimes if you want money she doesn’t give it.

I: No? …

C: Because moms don’t want to.

I: No, okay.

C: Do you want to?

I: Well, maybe that can vary. What else can you tell me about your mom?

C: [Stands up, gets some toys, and begins to play.] (Boy, 6 years old)

### Parent as Trauma Trigger

For some of the children the very request to reflect on the abused parent seemed to work as a trigger for trauma-influenced reactions and strategies. These children seemed overwhelmed; their accounts changed from adequate descriptions of more neutral matters to clearly trauma-influenced reactions when asked about the parent. They responded with heightened arousal, lost their concentration, and became dissociative, disorganized, driven, or overwhelmed. These children’s access to inner representations of the abused parent seemed blocked.

#### Avoidance

The children who reacted with avoidance demonstrated different strategies. Some actively refused to answer the questions, while some showed a slightly idealized, easy-going, or numb attitude. Some children simply changed the subject and started to talk about or do something else, and some kept themselves occupied with describing neutral concrete matters.

I: Can you tell me about your mom?

C: … hmm … nothing.

I: What is she like?

C: Good.

I: Okay … in what way … what does she do …?

C: Um … in preschool … [starts telling about something he has done in preschool]. (Boy, 6 years old)

A few of the children responded to a question about the abused parent by starting to talk about the perpetrator instead. These children seemed to be more able to think and talk about the abuser than about the abused.

I: Can you tell me about your mom?

C: She, um … I have to think …

I: That’s okay.

C: What was the other thing I should think about?

I: I asked if you could tell a little bit about your mom …

C: No, the other thing.

I: No … that was what I asked …

C: No, another question I had to think about.

I: Well, I don’t really know about that …

C: Then …

I: Let’s take that one for now; tell me a little bit about your mom, what is she like?

C: I said that I have to think about that one … but I can tell you about Dad! (Girl, 6 years old)

#### Breakthrough of Intrusive Memories and Thoughts

Some children seemed overwhelmed by their exposure to violence and vulnerability and their capacity to reflect seemed blocked or reduced. Others spontaneously described specific traumatic experiences.

I: Okay, I was thinking you could … can you tell me about you and your mom?

C: Um, Dad and Mom when they were in the car and leaving, then Dad just took Mom’s … like that … Dad started to throw … but, on, out, then did, and it didn’t come, it came on the floor …

I: On the floor …

C: Yes, in the car, and then Mom took it.

I: Mom took it …

C: Yes and then Dad just broke the hands …

I: Of?

C: Mom.

I: Okay … that hurt her …

C: Yes.

I: Were you there at that time?

C: Yes, and my sister. (Boy, 6 years old)

## Discussion

The present study aimed to expand knowledge of how young children who have witnessed IPV describe their abused parent. We believe that the findings can augment our understanding of the mechanisms of lived trauma, improve our awareness of the current psychological health and behavior of children who have been exposed to IPV, and illuminate the needs of these children. In many cases, the children demonstrated a capacity to talk and reflect about their parent in a nuanced and coherent manner. Other children’s descriptions were more deficient and incoherent, while for some simply being asked about the parent, with no mention of vulnerability or any trauma experienced, triggered trauma reactions. The children’s accounts of their experiences of their parent reflect the impact of IPV on parenting that has been described elsewhere; the coherent accounts may correspond with compensating mothers and the deficient with abdicated or impulsive and punitive mothers (George and Solomon 2008; Levendosky et al. 2003).

The development of a child’s capacities to mentalize and reflect is closely dependent on stimulation and the child’s experiences in the caregiving relationship (Fonagy et al. 2002; Ford 2009; Siegel 2012). Intimate partner violence has been associated with negative effects on parenting capacities, including diminished availability and reciprocity in the relationship, which in turn risk deficient development of mentalizing and affect-regulation capacities in the children (Schore 2013; Sroufe 2005). It is noteworthy that the interviewed children often demonstrated age-adequate abilities to reflect on the parent in an integrated manner, describing the parent as a person with resources and shortcomings, and the relationship as one of warmth, security, and belonging, with aspects of restriction, socializing, distance, and separation. Given the length and extent of the violence and exposure the families had lived through, it is notable that most of the children demonstrated these mentalizing abilities. This finding may relate to resilience in the children or perhaps their access to complimentary attachment relationships. It may also indicate that the abused parents had managed to offer their children sufficient reciprocity, predictability, and security. The results further contradict the position that young children are not able to contribute as informants about their own experiences and their relations with their parents.

Throughout childhood, during the process of psychological, cognitive, and social development, access to a caregiver as a secure base and safe haven is important (Bowlby 1980; Zeanah et al. 2011). For babies and very young children this access needs to be physical; later on children can increasingly use inner working models for affect-regulation and social relations. The present study provides reason to assume that for some of the children who gave deficient accounts of their parent the caregiver had not been, or was not at the time, sufficiently available and predictable to the child to develop emotional regulation and inner working models to be used in future relations (Levendosky et al. 2011; Schore and Schore 2008; Sroufe 2005).

Internal working models can be detected in the way the parent and the relationship are described and reflected upon (Main 2000). Traumatic experiences during childhood risk impairing the development of a reflective capacity and later reminders of trauma have been shown to block the capacity to mentalize (Fonagy et al. 2002; Ogden et al. 2006; Siegel 2012). The theme “Coherent accounts of the parent,” which contains rich and varied accounts, may include children with access to integrated working models of the parent and a capacity to reflect on different aspects of the parent and the child-parent relationship. Within the theme “Deficient accounts of the parent,” children display less coherence and these accounts may illustrate flat or shattered working models of the parent. Finally, the theme “Parent as trauma trigger” illustrates how children can become overwhelmed by being asked about the parent and shows how trauma reminders can block a child’s access to inner representations of the abused parent and the capacity to reflect and mentalize.

Special consideration should be paid to the children for whom talking about the abused parent seemed to trigger reactions of avoidance or the intrusion of traumatic memories. It is notable that the children were not asked about exposure of violence during the interviews, simply talking and thinking about the parent was triggering to them. It may be that these children’s internal working models of their primary caregiver are associated with fright: an association that may cause the children to strive to keep emotional distance or to control their fear through aggressiveness or role reversal in the relationship. Children who have witnessed IPV have been shown elsewhere to risk developing controlling attachment patterns, such as controlling punitive behavior or compulsive caregiving (George and Solomon 2008).

One possible effect of trauma is hypervigilance and sensitivity to reminders that may trigger trauma reactions in new situations long after the original trauma has ceased (Ogden et al. 2006; Siegel 2012). The results of this study indicate that not only the perpetrator but also the abused parent can serve as a reminder of trauma. These reminders can keep the child aroused and hyper-vigilant and can strengthen tendencies of avoidance even after the violence has ceased and even without contact with the perpetrator. A constant affective state of heightened arousal may negatively affect the psychological well-being and learning possibilities of the child (Siegel 2012). In the aftermath of IPV, the need to be hypervigilant and ready to protect oneself in the face of trauma-triggering stimuli risks hindering some children from fully recovering and thriving within the relationship with the abused parent, spontaneously or in treatment. Because the child may associate closeness with the caregiver with exposure to trauma reactions, there is a risk the child may tend to avoid or in other ways control the emotional closeness with the caregiver and thus become a co-creator of emotional distance between child and caregiver. This could have a severely negative impact on the child’s development and may lead to avoidance of intimacy in other relations as well.

### Clinical Implications

Treatment for children exposed to IPV that includes the caregiver and targets the relation between the child and the abused parent has been shown to be effective (Stover et al. 2009). Relational and dyadic treatment typically builds on identified strengths and resources in the child-parent relationship and focuses on changing problematic aspects. The results of the present study suggest that in treating children exposed to IPV it is necessary to address the possibility that not only the perpetrator, but also the abused parent (or aspects of the relationship with the abused parent), may trigger trauma reactions. This could result in the child avoiding the very relationship in which therapists and other professionals hope the child will find recovery and future resources for development. Participation in treatment may also result in the child being triggered and aroused. Thorough interviews with children can contribute to our understanding of the child-parent relationship and thus help to adjust and improve interventions.

One challenge in treatment will be to help turn a relationship that at times is associated with danger into a calm and secure source of new experiences of trust, nurturance, and protection in treatment and in everyday life. In this work it will be necessary to pay attention to and recognize signs of trauma reaction within the relationship and to address this in treatment through psycho-educational elements, affect-regulation strategies, or trauma processing. Such interventions will aim at helping children and caregivers to understand and make meaning of their former and current experiences and give space and possibility for new shared relational experiences. This approach will also entail the necessity of addressing parental needs of emotional and educative support to diminish arousal and trauma reactions in the parent in order to augment parental capacities and parental availability to the child. Treatment that does not target emotional regulation and the complexity of the parent-child relationship in the aftermath of IPV risks aggravating symptoms as well as hindering recovery and learning rather than promoting enhanced psychological health.

### Methodological Implications

The result of the present study demonstrates that children can participate in and contribute to methodologically sound research on matters that concern them. This is important as it strengthens the voices of children in accord with the UN Convention on the Rights of the Child and it adds necessary building blocks to complement existing research in the field. However, special issues of reliability and ethics, including developmental implications on children’s reflective capacity and methodological considerations on the imbalance in power between interviewer and child, must be addressed when interviewing vulnerable children. The results of the present study further indicate that some children are highly vulnerable and post-traumatic reactions are easily triggered. Actions must be taken to ensure these children’s sense of safety in the interview situation, to help them regulate their participation, and to allow access to support from the caregiver or from professionals when needed.

#### Limitations and Needs for Future Research

This study aimed to expand knowledge of how children who have witnessed IPV view their abused parent and their relationship to that parent. The research design, however, does not permit generalization of the results to groups and settings other than the children interviewed. These results must be considered in the context of earlier and future research to reveal to what extent the themes found are specific or shared with others.

Factors that might influence the quality of the interviews, such as the interviewer’s ability or concern for the children’s well-being, the location, the time of the day, etc., must also be considered. The degree of variation in the sample was restricted because the children in the study all lived in urban areas, were recruited from agencies providing treatment interventions, and had been identified as being in need of intervention. These similarities in the sample may diminish the extent to which these results can be applied to other children exposed to IPV in their families. The fact that some of the children had experienced physical violence against themselves and some, but not all were still in contact with the perpetrator may also have influenced the results. Furthermore, it is unknown to what extent other traumas and adverse experiences influenced the parent child relationship. However, this variety reflects the diversity of living conditions among children exposed to IPV even when they share the similarities mentioned above.

To further expand knowledge about the experiences and needs of children exposed to IPV there is a need for studies that take into account the perspectives of children. Research into the experiences of children at different ages, children living in foster care, or children with experience of other kinds or trauma would shed additional light on the results from this study.

## Conclusions

Children in early and middle childhood who have witnessed IPV are able to reflect upon and talk about their abused parent and their relationship with that parent. The children were shown to have both capacities and difficulties in reflecting upon the abused parent, indicating that the children may have both integrated and deficient or blocked internal representations of the parent in the aftermath of IPV. The awareness of this variety and the possibility that the parent may serve as a trauma trigger will affect theory about the consequences of IPV and clinical practice in designing and performing interventions for children exposed to IPV.
